# PLCβ1-SHP-2 complex, PLCβ1 tyrosine dephosphorylation and SHP-2 phosphatase activity: a new part of Angiotensin II signaling?

**DOI:** 10.1186/1423-0127-18-38

**Published:** 2011-06-13

**Authors:** Lorenzo A Calò, Luciana Bordin, Paul A Davis, Elisa Pagnin, Lucia Dal Maso, Gian Paolo Rossi, Achille C Pessina, Giulio Clari

**Affiliations:** 1 Department of Clinical and Experimental Medicine, Clinica Medica 4 University of Padova, School of Medicine, Italy; 2 Department of Biological Chemistry, University of Padova, School of Medicine, Italy; 3 Department of Nutrition, University of California, Davis, USA

**Keywords:** Angiotensin II signaling, SHP-2, PLCβ1, SHP-2-PLCβ1 complex

## Abstract

**Background:**

Angiotensin II (Ang II) signaling occurs via two major receptors which activate non-receptor tyrosin kinases that then interact with protein tyrosin-phosphatases (PTPs) to regulate cell function. SHP-2 is one such important PTP that also functions as an adaptor to promote downstream signaling pathway. Its role in Ang II signaling remains to be clarified.

**Results:**

Using cultured normal human fibroblasts, immunoprecipitation and western blots, we show for the first time that SHP-2 and PLCβ1 are present as a preformed complex. Complex PLCβ1 is tyr-phosphorylated basally and Ang II increased SHP-2-PLCβ1 complexes and caused complex associated PLCβ1 tyr-phosphorylation to decline while complex associated SHP-2's tyr-phosphorylation increased and did so via the Ang II type 1 receptors as shown by Ang II type 1 receptor blocker losartan's effects. Moreover, Ang II induced both increased complex phosphatase activity and decreased complex associated PLCβ1 tyr-phosphorylation, the latter response required regulator of G protein signaling (RGS)-2.

**Conclusions:**

Ang II signals are shown for the first time to involve a preformed SHP-2-PLCβ1 complex. Changes in the complex's PLCβ1 tyr-phosphorylation and SHP-2's tyr-phosphorylation as well as SHP-2-PLCβ1 complex formation are the result of Ang II type 1 receptor activation with changes in complex associated PLCβ1 tyr-phosphorylation requiring RGS-2. These findings might significantly expand the number and complexity of Ang II signaling pathways. Further studies are needed to delineate the role/s of this complex in the Ang II signaling system.

## Background

Angiotensin II (Ang II) is a major regulator of a broad spectrum of important biological processes ranging from vasoconstriction to inflammatory processes including atherosclerosis and vascular ageing, which proceeds, in part, via phosphoinositide-specific phospholipase C (PLC) generated second messengers [1-4]. Ang II type 1 receptors couple first to PLCβ1 via Gαq/11βγ and Gαq/12 βγ and then to PLCγ via tyrosine kinase activity [5]. Ang II also induces phosphorylation of growth signaling kinases by redox-sensitive regulation of protein tyrosine phosphatases (PTPs) [6] via oxidation/inactivation and blunted phosphorylation of the PTP, SHP-2. Ali et al [7] demonstrated that Ang II induces SHP-2 tyrosine phosphorylation and activation of its phosphatase activity. In addition to its phosphatase activity, SHP-2 appears to function as a molecular adaptor as shown by Ali et al's report of a SHP-2 IRS complex [7] as well as its adaptor function being inferred from the substantial differences noted between dominant negative mutant SHP-2 (mild phenotypes [8]) and SHP-2 knockout (severe phenotypes [9,10]). Finally, SHP-2's participation in Ang II signaling has also been recently revealed through the demonstration of its central role in the regulation of RhoA-Rho kinase pathway's activation [11], another important pathway downstream of Ang II type 1 receptor stimulation which, when activated, ultimately leads to both vasoconstriction and cardiovascular remodeling [12,13].

The previous report of a complex involving SHP-2 suggests that SHP-2 may function as part of a complex in other pathways. The concept of and the role(s) for complex formation has gained increasing attention as a means to direct signals toward a particular pathway along with reducing the likelihood of cross-talk by Golebiewska et al [14]. For example, they have shown that during Gαq signaling, Gαq, rather than selecting a specific effector during stimulation, functions via separate pools of Gαq-effector complexes [14].

During the course of investigating Ang II signaling in our well characterized "in vivo" human model of altered Ang II long term signaling and vascular tone control, Bartter's and Gitelman's syndromes [13,15-19], we have produced findings suggesting the presence of another complex involving SHP-2. This report represents our initial efforts to confirm and further investigate the characteristics of SHP-2-PLCβ1 interaction as preformed complex and its interaction with selected aspects of Ang II signaling. The current study was undertaken in normal human fibroblasts and employed specific antibodies to immunoprecipitate and then characterize the resulting immunoprecipitates, i.e. anti PLCβ1 or anti SHP-2 immunoprecipitates of cultured fibroblast cell lysates were probed after western blotting using anti PLCβ1, anti SHP-2 and anti phospho tyrosine antibodies. In addition, we probed Ang II signaling processes related to this complex by assessing the effects of losartan, an Ang II type 1 receptor blocker, as well as by altering, via its silencing, the levels of the regulator of G protein signaling 2 (RGS-2), a key control element of Ang II signaling [20,21].

## Results

The effect of Ang II on PLCβ1 and SHP-2 in human skin fibroblasts was examined using cultured cells incubated with or without Ang II (100 nM) for 1 h. The effect of Ang II was examined by probing Western blots (analysed by 8% SDS/PAGE gels) of cell lysates immunoprecipitated with either anti PLCβ1 antibody or anti-SHP-2 antibody. The figures are the results of representative experiments. antibody. The figures are the results of representative experiments.

**Figure 1:**
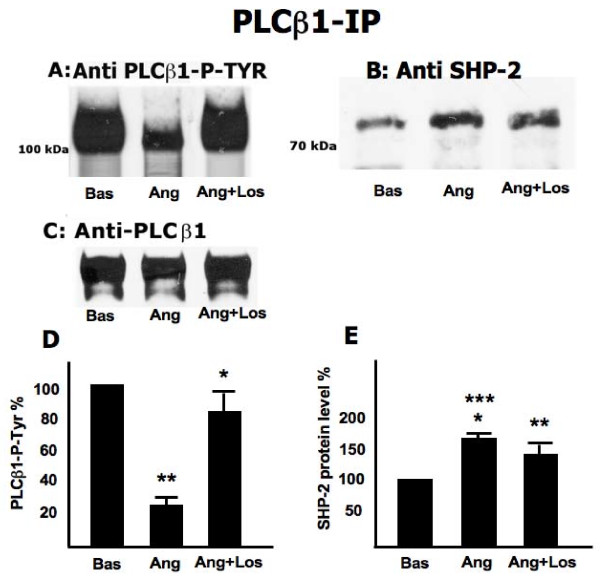
**Effect of Ang II and losartan on PLCβ1 Tyr-phosphorylation and SHP-2 content**. Fibroblasts, cultured in absence or presence of Ang II (100 nM) and Ang II (100 nM) plus losartan (100 μM), were scraped and extracted with buffer C (see methods). Total cell lysate (200 μg) were immunoprecipitated with anti-PLCβ1 antibody. Immunoprecipitates were subjected to Western blotting (analysed by 10% SDS/PAGE gels) and immunorevealed with mouse-anti-PLCβ1 P-Tyr (A) and rabbit-anti-SHP-2 (B) antibodies, before being stripped and immunorevealed with anti-PLCβ1 (mouse) (C). The figure is representative of five separate experiments carried out in duplicate. Panels D and E present the percent change relative to unstimulated cells ± SD (N = 5 experiments) for panel A and B respectively. Panel D: **: p < 0.0001 vs Basal; *: p = 0.003 vs Ang. Panel E: ***: p < 0.0001 vs Basal; *: p = 0.006 vs Ang+Los; **: p = 0.002 vs Basal.

Figure 1A reveals a strong band upon probing the PLCβ1 immunoprecipitate of nonstimulated cells with anti PLCβ1 phospho tyrosine, which declines (-74.43%) when cells are treated with Ang II and is restored (-17.6% of nonstim) when cells are treated with Ang II plus losartan. Figure 1B shows the presence of SHP-2 in the PLCβ1 immunoprecipitate in the unstimulated state demonstrating the formation of a complex between SHP-2 and PLCβ1. Upon treatment with Ang II, the level of SHP-2 protein in the PLCβ1 immunoprecipitate increased (+63.7%) which then declines (+34.8%) when cells are treated with Ang II plus losartan. The absence of any difference when probing the PLCβ1 immunoprecipitate with anti PLCβ1 demonstrates that the decline seen in upon Ang II treatment (Figure 1A) was only due to changes in phosphorylation. Figures 1D and 1E present the % change relative to unstimulated cells ± SD (N = 5 experiments) for 1A and 1B respectively.

**Figure 2:**
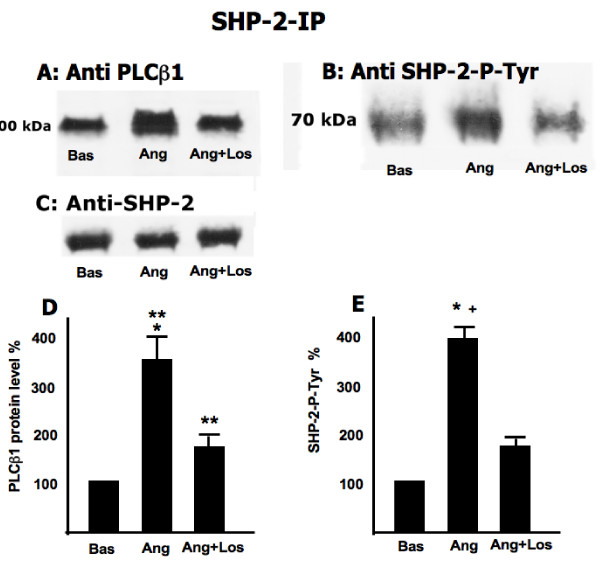
**Effect of Ang II on SHP-2 association with PLCβ1 and SHP-2 Tyr-phosphorylation**. Total cell lysate (200 μg) were immunoprecipitated with anti-SHP-2 antibody. Immunoprecipitates were subjected to Western blotting (analysed by 8% SDS/PAGE gels) and immunorevealed with mouse-anti-PLCβ1 (A), rabbit-anti-phospho tyrosine SHP-2 (B) antibodies, rabbit anti SHP2(C). The figure is representative of five separate experiments carried out in duplicate. Panels D and E present the percent change relative to unstimulated cells and SD (N = 5 experiments) for panel A and B respectively. Panel D: **: p < 0.0001 vs Basal; *: p = 0.001 vs Ang+Los Panel E: *: p < 0.0001 vs Basal; **+**: p < 0.0001 vs Ang+Los.

Figure 2B shows a band upon probing the SHP-2 immunoprecipitate of nonstimulated cells with anti SHP-2 phospho tyrosine which increases (+345.6%) when cells are treated with Ang II and declines (+179% of nonstimulated cells) when cells are treated with Ang II plus losartan. Figure 2A reveals the presence of PLCβ1 protein in the SHP-2 immunoprecipitate in the unstimulated state demonstrating the formation of a complex between SHP-2 and PLCβ1. Upon treatment with Ang II, the level of PLCβ1 protein in the SHP-2 immunoprecipitate increased (+393.8%) which then declines (+112%) when cells are treated with Ang II plus losartan. Figures 2D and 2E present the % change relative to unstimulated cells and SD (N = 5 experiments) for 2A and 2B respectively. The absence of any difference when probing the anti SHP-2 immunoprecipitate with anti SHP-2 demonstrates that the increase seen in upon Ang II treatment (Figure 2B) was only due to changes in phosphorylation.

**Figure 3:**
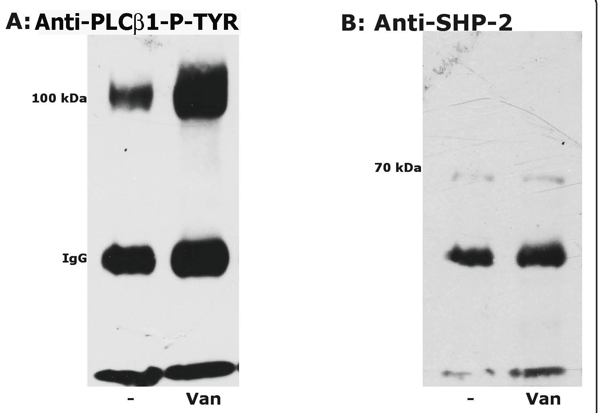
**Effect of vanadate on PLCβ1-Tyr-phosphorylation and SHP-2 association**. Cells were cultured in the absence or presence of vanadate (1 mM) and total cell lysates (200 μg) were immunoprecipitated with anti-PLCβ1 antibody. Immunoprecipitates were subjected to Western blotting (analysed by 10% SDS/PAGE gels) and immunorevealed with mouse-anti-P-Tyr (A) and rabbit-anti-SHP-2 (B) antibodies. The figure is representative of three different and separate experiments.

Figure 3 presents the results of incubation in the presence of vanadate. Figure 3A shows that the level of PLCβ1 phospho tyrosine increases upon phosphatase inhibition.

Figure 3B shows that the amount of SHP-2 protein does not change upon incubation with vanadate.

**Figure 4:**
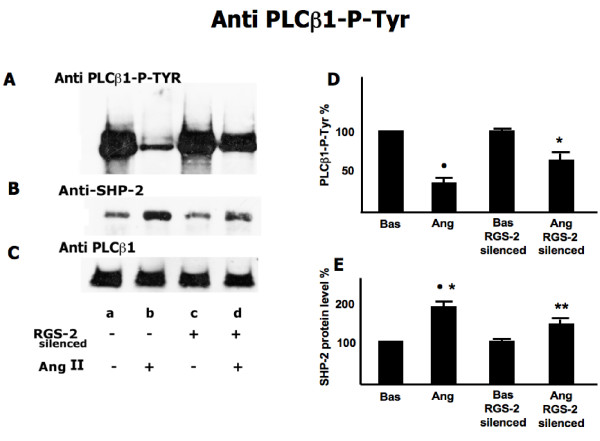
**Effect of Ang II on PLCβ1-Tyr-phosphorylation and SHP-2 association in RGS-2 silenced and not silenced cells**. RGS-2 not silenced (lanes a, b) and silenced (lanes c, d) fibroblasts were incubated with Ang II (lanes b, d,) or with vehicle (lane a, c) for 1 hour as described in the Methods. Immunocomplexes were isolated and analyzed as described in methods. Panel A is PLCβ1 Phospho-Tyrosine levels, panel B is SHP-2 protein levels and panel c is PLCβ1 protein levels. The figure is representative of five separate experiments carried out in duplicate. Panels D and E present the percent change relative to unstimulated cells and SD (N = 5 experiments) for panel A and B respectively. Panel D: •: p < 0.0001 vs Basal; *: p < 0.0001 vs Ang RGS-2 Silenced; **: p < 0.0001 vs Basal RGS-2 Silenced. Panel E: •: p < 0.0001 vs Basal; *: p = 0.04 vs Ang RGS-2 Silenced; **: p = 0.001 vs Bas RGS-2 Silenced.

Figure 4 shows the effects of RGS-2 silencing on the protein levels of both SHP-2 and PLCβ1 as well as the phosphorylation of PLCβ1. Figure 4A shows that RGS-2 silencing abrogates the dephosporylation of PLCβ1 phospho tyrosine induced by Ang II (-69%). Figure 4B shows that silencing of RGS-2 reduces the increase of SHP-2 protein in the PLCβ1 immunoprecipitate when cells are treated with Ang II. Figure 4C shows that PLCβ1 protein level in PLCβ1 immunoprecipitate is unaffected by RGS-2 silencing. Figures 4D and 4E present the % change relative to RGS-2 intact and unstimulated cells ± SD (N = 5 experiments) for 4A and 4B respectively

The phosphatase activity of the SHP-2 immunoprecipitates of cells significantly increased in normotensive healthy subject cells with Ang II compared to those without Ang II (1.55 ± 0.2 versus 1.0 ± 0.2 nanomoles per min per 200 mg cell protein immunoprecipitate, p < 0.005).

## Discussion

The present study on Ang II signaling in normal human fibroblasts has produced the first description, to our knowledge, of the presence of a SHP-2-PLCβ1complex that responds to Ang II signaling associated events. The presence of a SHP-2-PLCβ1 complex in fibroblast from normotensive healthy subjects was demonstrated via immunoprecipitates obtained by incubating with either anti PLCβ1 or anti SHP-2 (Figure 1 and 2). The relationship of this complex to Ang II signaling was demonstrated by the fact that the degree of phosphorylation of both PLCβ1 and SHP-2, was reciprocally affected by Ang II. Incubation with Ang II caused the dephosphorylation of PLCβ1 and the phosphorylation of SHP-2. The effect of Ang II on these was further demonstrated by the blocking of these changes found by incubation in the presence of losartan. Moreover the linkage of the SHP-2-PLCβ1 complex to Ang II signaling events is further strengthened by the effect of RGS-2 silencing which blocked Ang II induced changes in the phosphorylation status of the complex proteins. In addition, Ang II incubation led to an increase in total immunoprecipitable phosphatase activity. That SHP-2 may act as a phosphatase with respect to PLCβ1 is suggested by the increased PLCβ1 phosphorylation in immunoprecipitation experiments in the presence of vanadate to inhibit phosphatase activity. The absence of changes upon Ang II treatment in the amount of PLCβ1 protein isolated by anti PLCβ1 immunoprecipitation demonstrates that the altered PLCβ1 tyrosine phosphorylation (Figure 1A) found was due to changes in PLCβ1 tyrosine phosphorylation and not due to changes in protein amount. However, this does not appear to be the case with respect to SHP-2 immunoprecipitation as the protein levels of PLCβ1 differed among the treatments. This may be the result of differences in free versus complex bound SHP-2 levels in the cells.

SHP-2, participates in multiple signal transduction cascades, including the Ras-Raf-MAP kinase, JAK/STAT, PI3K/Akt, NF-κB, and NFAT pathways [22-24] and accumulating evidence suggests that SHP-2 also functions as an adaptor/scaffolding. In fact Wang et al [25] showed that SHP-2 functions in Interleukin-1 signaling as a part of a complex that was dependent on focal adhesions, which are enriched with tyrosine kinases and SHP-2. That SHP-2 functions as an adaptor/scaffolding is also suggested by the disparate nature of the effects of overexpression of mutated, catalytically inactive SHP-2, as compared to SHP-2 knockout [22]. Using this model, Bregeon and coworkers have recently demonstrated a central role of SHP-2 activity as a scaffold protein in the regulation of RhoA-Rho kinase pathway's activation [11]. In fact, they found that SHP-2 is necessary to allow the association of the tyrosine kinase c-Abl with p190A, a RhoA activating GTPase and the c-Abl-mediated p190A phosphorylation to maintain basal p190A activation and consequently a low RhoA-Rho kinase activity. In addition, this study reports that SHP-2 phosphatase activity itself is necessary to promote p190A dephosphorylation and inhibition in response to Ang II via Ang II type 1 receptor activation [11], therefore activating or prolonging RhoA-Rho kinase pathway's activity. On the other hand, Ang II type 2 receptor stimulation seems to be involved in the inhibition of SHP-2 phosphatase activity as shown by the greater effect on p190A dephosphorylation in the presence of Ang II type 2 receptor antagonist, while Ang II-induced p190A-dephosphorylation was abolished in the presence of the Ang II type 1 receptor inhibitor losartan [11].

The current study identifies a preformed SHP-2-PLC β1 complex as a part of Ang II signaling which strengthens the concept that preformed complexes are involved in cell signaling systems. These complexes have been suggested to function to direct signals toward a particular pathway along with reducing the likelihood of cross-talk [14]. For example, it was reported that during Gαq signaling, Gαq, rather than selecting a specific effector during stimulation, functions via separate pools of Gαq-effector complexes [14]. Similarly the SHP-2-PLCβ1 complex identified in the present study may function in cardiac hypertrophy via Ang II type 1 receptor stimulation as PLCβ1 has been implicated by Filtz et al [26].

## Conclusions

The identification of a SHP-2-PLCβ1preformed complex that responds to Ang II as shown in this study is an important first step but the role of this complex in the Ang II signaling remains to be delineated. We are aware that this is a limitation of the present study, however we think that the identification of this complex and its response to Ang II merits to be reported waiting for the results of further experiments specifically performed to clarify its role in the Ang II signaling. To this purpose, one approach to understanding the role of SHP-2-PLCβ1 complex is to assess its status in two systems with contrasting Ang II signaling. A comparison of the complex's levels and behavior in Bartter's and Gitelman's syndromes, a human model of blunted Ang II signaling system and RhoA-Rho kinase pathway [13,15-18] and activation of Ang II type 2 receptor signaling [19] to the complex's levels and behavior in hypertensive patients, which have Ang II signaling system and RhoA-Rho kinase pathways, biochemical, molecular and clinical features opposite to those of the Bartter's and Gitelman's patients [16], might provide insight into the complex's role in Ang II signaling. These studies are ongoing in our laboratory and their results along with those from other potential studies examining aspects such as the respective SHP-2- and PLCβ1 binding site characteristics, likely will significantly expand the number and complexity of the signaling pathways through which Ang II signals and thereby might provide new potential targets of therapy for diseases such as hypertension, diabetes and cardiovascular disease, in which Ang II plays a major role.

## Methods

Anti-P-Tyr and anti-PLCβ1monoclonal antibodies were purchased from Biosource (Prodotti Gianni, Milano, Italy) and Upstate (Lake Placid, NY, USA), respectively while rabbit anti-SHP-2 (C-18) polyclonal antibody was from Santa Cruz Biotechnology (CA, USA). Protease inhibitor cocktail was obtained from Roche Diagnostic (Indianapolis, IN, USA). Anti-mouse and anti-rabbit secondary antibodies conjugated with horseradish peroxidase (HRP) were from (Calbiochem (Darmstadt, Germany).

### Cell Culture

Skin fibroblasts from 6 healthy subjects from the staff of the Department of Clinical and Experimental Medicine at the University of Padova, who gave their informed consent, were obtained via biopsy and individually cultured in F-10 HAM medium with 10% fetal bovine serum, 100 U/ml penicillin, 100 mg/ml streptomycin and 4 mmol/l glutamine, as previously described [19,27,28] and used after the third passage. To assess the effects of Ang II, cells were incubated with 100 nM Ang II for 1 hour. This concentration was chosen, since it was clearly seen to induce Ang II signaling in previous reports [19,29,30]. To assess the effects of phosphatase activity on protein phosphorylation, cells were incubated with 1 mM vanadate overnight. To examine the effect of Ang II type 1 receptor signaling blockade, cells were preincubated for 30 min with 100 μM losartan and then treated as described above. This concentration was also chosen based on a previous report [19].

### Immunoprecipitation

Anti-SHP-2 and Anti PLCβ1 immunoprecipitation was done using confluent cells. These were scraped, washed in buffer and extracted (1 h at 4°C with buffer C (20 mM Tris-HCl, pH 7.5, 10% glycerol, 1% Nonidet-P-40, 1 mM EDTA, 150 mM NaCl, 1 mM sodium orthovanadate, protease inhibitor cocktail). After centrifugation, 200 μg of supernatant protein were diluted 1:1 in 20 mM Tris-HCl, pH 7.5, containing 1 mM sodium orthovanadate and protease inhibitor cocktail, precleared with protein A-Sepharose, and anti-SHP-2 or anti PLCβ1antibodies bound to protein A-Sepharose were added at 4°C. This was then incubated overnight, immunoprecipitates were washed 3× in buffer D (25 mM imidazole, pH 7.0, 1 mM EDTA, 0.02% NaN3, 10% glycerol, 10 mM B-mercaptoethanol, 10 mg/ml leupeptin, 50 mM PMSF), resuspended and then submitted to gel electrophoresis (SDS-PAGE; 8% or 10% gels), transferred by blotting to nitrocellulose membranes and immunostained with the appropriate antibodies/second antibodies.

### Phosphatase Activity

Phosphatase activity was measured at 30°C using nitrophenyl phosphate (pNPP) (10 mM pNPP as substrate in 100 mM tris-HCl (pH 7.4), 150 mM NaCl, 1 mM EDTA, 1 mM 2-mercaptoethanol, and PTPase immunoprecipitates from 200 mg cell-protein content in Buffer D). After 10 min at 30°C, the reaction was quenched with 950 μl of 1 M NaOH. Absorbance at 405 nm was measured and in all cases the substrate-to-product conversion was less than 5%. All the reagents were from Sigma (Milano, Italy). Results are expressed as nanomoles per minute per 200 mg cell protein immunoprecipitate.

### RGS-2 Silencing

RGS-2 gene silencing was done using chemically synthesized siRNA that mapped to exon 5 of RGS-2 gene (Silencer Pre-Designed siRNA, Ambion, Austin, USA) as previously described [27]. Fibroblasts (2 × 10^5 ^cells) were plated the day before transfection in 6-well plates in growth medium without antibiotics containing 10% FBS. On the day of transfection, siRNA was incubated with Lipofectamine 2000 diluted in OPTI-MEM I (Invitrogen, Carlsbad, USA) following manufacturer's instructions (Invitrogen, Carlsbad, USA). We have chosen for our experimental protocol RGS-2 siRNA mapping for exon 5 at a concentration of 50 nmol/l and transfected the oligos with Lipofectamine 2000 (4 mg/ml) as previously reported [27]. Following 20 min incubation at room temperature, the obtained complexes were added drop-wise onto the cells subcultured in replaced cell-culture medium. The cells were maintained in a 37°C incubator until analysis. The medium was changed to medium with no siRNA 12 h after transfection. Fluorescein-conjugated siRNA (Control (non-silencing), Fluorescein, Qiagen, Hilden, Germany) with no sequence identity for any human gene was used as negative control to exclude non-specific effects and to monitor the efficiency of transfection while GAPDH siRNA was used as positive control (Ambion, Austin, TX USA). Silencing was assessed by western blot and found to be 44% as previously reported [27]. Horseradish peroxidase (HRP)-conjugated (Amersham Pharmacia, Uppsala, Sweden) antibody was used as secondary antibody and visualized with chemiluminescence, which was captured on radiograph film. Exposed films were digitized by scanning densitometry and protein levels were calculated using National Institutes of Health (NIH) Image software (NIH, Bethesda, Maryland, USA). β actin was used as housekeeping gene and the ratios between RGS-2 and β actin western blot products were used as index of RGS-2 protein expression and expressed as densitometric arbitrary units.

### Statistical analysis

Data were evaluated statistically as normally distributed continuous variables and comparisons were performed using one-way ANOVA (Statistica, Statsoft Inc, Oklahoma City, OK, USA). Results with p < 0.05 were considered significant and data values are presented as mean±SD.

## Competing interests

The authors declare that they have no competing interests.

## Authors' contributions

LAC designed the experimental protocol and wrote the manuscript. LB contributed to design the experimental protocol, helped to drafting the manuscript and contributed to perform the experiments. PAD helped to design the experiments, contributed to drafting the manuscript and did the statistical analysis. EP and LDM performed the experiments. GPR, ACP and GC reviewed the manuscript. All authors read and approved the final version of the manuscript.
